# Determinants of premature rupture of membrane among pregnant women in Harar town, Eastern Ethiopia: A case-control study

**DOI:** 10.1016/j.heliyon.2023.e15445

**Published:** 2023-04-11

**Authors:** Addisu Getnet, Lemessa Oljira, Nega Assefa, Getahun Tiruye, Zerihun Figa

**Affiliations:** a Department of Midwifery, College of Health and Medical Sciences, Dilla University, Dilla, Ethiopia; b School of Public Health, College of Health and Medical Sciences, Haramaya University, Harar, Ethiopia; c School of Nursing and Midwifery, College of Health and Medical Sciences, Haramaya University, Harar, Ethiopia

## Abstract

**Introduction:**

Premature rupture of membrane is a disruption of fetal membrane followed by passage of watery fluid gush before the onset of labor any time beyond 28 weeks of gestation. It is a significant cause of perinatal morbidity and mortality. Many studies were conducted on the pre-labor rupture of membrane, yet limited evidence is available on its determinants. This study aimed to identify determinants of premature rupture of membrane among pregnant women in Harar Town, Eastern Ethiopia.

**Method:**

ology; Hospital-based unmatched case control study was conducted on 115 cases and 230 controls from 15th May to 15^th^ July 2021. The study was conducted on two public hospitals in Harar town. All cases admitted at the time of data collection were included until the required sample size was obtained and controls were selected by using simple random sampling among all non -cases. Data were collected using interviewer-guided semi-structured questionnaires. The data were entered into Epi-data version 3.1 and exported to SPSS version 22.0 for analysis. Binary logistic regression was used to identify determinants of the outcome and statistical significance declared at a p-value less than 0.05. Multicollinearity was checked and model fitness was assessed by Hosmer Lemeshow test.

**Results:**

Abnormal vaginal discharge [AOR 2.15 (2.53, 22.46), history of cesarean delivery [AOR 2.06 (1.11, 6.78)], history of premature rupture of membrane [AOR 4.62 (2.06, 11.52)] and history of abortion [AOR 2.81 (1.04, 6.23)] increase the odds of the outcome.

**Conclusion:**

Bad obstetric histories in the current and previous pregnancies are related to premature rupture of membrane. Therefore, it is recommended that health care providers should take attention to women with past and current pregnancy complications.

## 1 Introduction

Rupture of the fetal membrane before the onset of labor any time beyond the 28th weeks of gestation is said to be premature rupture of membrane [1]. It is characterized by the passage of watery discharge per vaginum, which is a painless gush or slow leak. The diagnosis is usually made through visualization of cervical free flow of fluid or pooling in the vaginal fornix upon sterile speculum examination [1,2].

The incidence of premature rupture of membrane (PROM) is 5–10% within all pregnancies. Rupture occurs in 68% of term pregnancies however, 92% undergo labor within 24 h [3]. PROM complicates 8–10% of pregnancies and results in adverse maternal and neonatal outcomes [4]. Intra amniotic infection is a major maternal complication of PROM, which occurs in about 31.5% of mothers who had PROM [5]. Newborns delivered from mothers with PROM are at risk of sepsis, prematurity, and birth asphyxia which are the leading causes of neonatal death in developing countries. One -third of preterm births are associated with PROM [6,7].

Different factors contribute to the occurrence of PROM. Genital infections, smoking, hypertensive disorders during pregnancy, previous history of cesarean delivery, previous history of abortion, and previous history of PROM are risk factors for the occurrence of PROM [2,8,9].

Different strategies were developed to reduce the adverse outcomes of PROM. Hospital admission and management of cases expectantly were the recommended practices to lower perinatal complications of PROM [10]. In Ethiopia antibiotics for PROM is one of the packages in the national newborn and child survival strategy document set to decrease adverse perinatal outcomes [11].

Studies have indicated PROM as a risk factor for preterm birth and neonatal infections, which are the major causes of early and late neonatal death in Ethiopia. Though studies were conducted on PROM; data on its determinants were scarce in the study area [5,7,12]. Therefore, this study was aimed to identify determinants of premature rupture of membrane among pregnant women in Harar town public hospitals, Eastern Ethiopia.

## 2 Methods and materials

### 2.1 Study setting and design

This unmatched case-control study was conducted in Harar town public hospitals, eastern Ethiopia from May to July 2021. The two public hospitals in the town are Hiwot Fana Specialized University hospital and Jugol hospital. In Hiwot Fana Specialized University Hospital, the labor and delivery unit admits 3500–5000 cases annually including referral cases from neighboring districts. Jugol hospital admits 2000–3500 labor and delivery cases annually.

### 2.2 Target population

All pregnant women in Harar town were the target population for whom the result of this study was generalized.

### 2.3 Source population

All pregnant women who were admitted to labor and delivery units in public hospitals of Harar town were the source population.

### 2.4 Study population

All pregnant women admitted to labor and delivery units in the selected hospitals during data collection time were included in the study. Cases and controls were included based on the criteria set in the case definition. Women with serious fetal or maternal deterioration need immediate delivery were excluded from the study.

Case definition of PROM: women who had experienced spontaneous rupture of membrane any time before the onset of labor as diagnosed by Physician/Midwife/Nurse using criteria set by American College of Obstetrics and Gynecology (ACOG) criteria. Physician/midwife/nurse made the diagnosis of PROM from the history of spontaneous passage of watery vaginal fluid that wet the perineum plus sterile speculum examination. The diagnosis of PROM at speculum examination was from visualization of fluid pooling in the fornix or free flow of fluid from the cervix.

**No PROM (controls):** Pregnant women admitted to the labor and delivery units who started labor with intact amniotic membrane were selected as controls.

## 3 Inclusion and exclusion criteria

### 3.1 Inclusion criteria

All pregnant women admitted to labor and delivery units with the diagnosis of PROM and all mothers who came for labor and delivery with no ruptured membrane at the onset of labor in selected hospitals were included in the study.

### 3.2 Exclusion criteria

Mothers with serious fetal or maternal deterioration need immediate delivery were excluded from the study.

#### 3.2.1 Sampling and sample size determination

The sample size was estimated by using EPI INFO version 7.2.1 statistical software using double population formula for an unmatched case-control study. By considering the proportion of history of previous cesarean delivery in controls 4.4% with adjusted odds ratio (AOR) 3.67 in the previous study done in Tigray, Ethiopia [8]. With the assumption of 95% confidence interval (CI): 80% power, controls to cases ratio 2:1 the sample size becomes 320. By adding a 10% non-response rate the final sample size became **351** (117 cases and 234 controls). The sample size was allocated for each hospital proportionally based on the average patient flow in the unit within two months. From the first hospital (Hiwot Fana Specialized University Hospital 75 cases and 150 controls were selected. From the second hospital (Jugol hospital), 42 cases and 84 controls were selected. All PROM cases during the data collection period were included as cases until the required sample size was obtained. Controls were selected by using a simple random sampling method. Two controls were drawn at random by lottery method from a list of controls admitted after the selected case was admitted to the same unit. Control selection started immediately after selecting each case.

## 4 Data collection methods

Data were collected through interviewer guided semi-structured questionnaire. Questionnaires were prepared in English language and translated into Amharic and Afan Oromo languages, then back to English by a translator to ensure consistency. The questionnaire contains open and closed ended questions with socio demographic characteristics, past obstetric and gynecologic factors, and current pregnancy factors-related questions. Mothers with the respective Medical Record Numbers were selected for interview. Before the interview, data collectors explained the aim, risks, possible benefits, and the right to refuse to participate in the study issues. After stating all, those women who were willing to sign the voluntary consent form were interviewed. Client card review was done for clinical abstraction including gestational age, maternal hemoglobin level, fetal presentation, and type of pregnancy.

### 4.1 Data quality control

Before data collection, a pretest was done in 5% of the samples from Dilchora hospital in Dire Dawa, one of the nearby towns. Before starting data collection the data collectors and the supervisors took training for one day on the tool, data collection methods, ethical issues, and purpose of the study. Supervisors had checked the completeness of data and consistency of the information in the sheet.

### 4.2 Ethical approval

The study was approved by the institutional health research ethical review committee (ref no: IHRERC 014/21) of college of health and medical sciences, Haramaya University. Informed consent was obtained from the participants after explaining the purpose of the study. The respondents were told as they have the right to respond fully or partially to the questionnaire. All the information given by the respondents was used for research purposes only and confidentiality was maintained.

### 4.3 Data processing and analysis

The collected data were coded, cleaned, and entered to EPI data version 3.1 and exported to SPSS version 22 for analysis. Descriptive data analysis was presented using numerical summary measures and displayed using tables. Binary logistic regression was used to do crude and adjusted analysis. The goodness of model fitness was checked by Hosmer-Lemeshow statistic which was insignificant which indicates that the model was fitted. Variables with a p-value less than 0.25 in the crude analysis were entered into an adjusted logistic regression model. For variables with more than two categories we considered the overall p value to undergo multivariable analysis. But in multi variable regression result we took the p value in the specific category to declare significant association. Multicollinearity was assessed by a variance inflation factor (VIF) and standard error and no multicollinearity was detected. The VIF score was less than 5 and declared no multicollinearity. Adjusted odds ratio with 95% confidence interval was estimated to identify the independent determinants of premature rupture of membrane. Finally, variables that show a level of p-value less than 0.05, in the final analysis, were declared as statistically significant.

## 5 Results

A total of 345 respondents were included. The mean age of cases and controls were 25.3 ± 6.09 SD (standard deviation) and 24.5 ± 4.75 SD respectively. In both cases and controls majority were Oromo by ethnicity and Muslim by religion. 95 (82.6%) of cases and 198 (86.1%) of controls belong to Oromo ethnicity (Table 1).

**Table 1:** Sociodemographic characteristics of participants in public hospitals of Harar Town, Eastern Ethiopia 2020 (n = 345; 115 cases and 230 controls).

Variables	Category	Cases (N, %)	Controls (N, %)
Age	<20 years	16 (13.9)	45 (19.6)
	≥20 years	99 (86.1)	185 (80.4)
Marital status	Married	109 (94.8)	223 (97)
	Single	2 (1.7)	1 (0.4)
	Others^a^	4 (3.5)	6 (2.6)
Religion	Muslim	92 (80)	198 (86.1)
	Orthodox	15 (13)	21 (9.1)
	Protestant	5 (4.4)	9 (3.9)
	Othersᵇ	3 (2.6)	2 (0.9)
Ethnicity	Oromo	95 (82.6)	198 (86.1)
	Amhara	13 (11.3)	15 (6.5)
	Harari	2 (1.7)	6 (2.6)
	Othersͨ	5 (4.3)	11 (4.8)
Occupation	Housewife	92 (80)	190 (82.6)
	Gov't Employee	11 (9.6)	16 (7.0)
	Merchant	7 (6.1)	15 (6.5)
	Othersᵈ	5 (4.3)	9 (3.9)
Educational status	No formal education	53 (46.1)	107 (46.5)
	Formal education	62 (53.9)	123 (53.5)
Monthly income	<2500ETB	49 (42.6)	105 (45.7)
	2500-4999ETB	45 (39.1)	94 (40.9)
	>4999ETB	21 (18.3)	31 (13.5)
Residence	Urban	66 (57.4)	127 (55.2)
	Rural	49 (42.6)	103 (44.8)

a divorced, widowed, separated ᵇcatholic, Jehovah ᶜ Gurage, Silte, Tigray ᵈ student, farmer.

Half of the cases 42 (50%) and 36 (22.5%) of controls had a history of abortion in prior pregnancies. The proportion of women who had a history of cesarean section is higher among cases (21.4%) compared to controls. History of preterm birth was found in19 (22.6%) of cases and 17 (10.6%) of controls (Table 2).

**Table 2:** Past obstetric and gynecologic characteristics of participants in public hospitals of Harar Town, Eastern Ethiopia 2020 (n = 345; 115 cases and 230 controls).

Variables	Category	Cases (N, %)	Controls (N, %)
Prior pregnancy history	YES	84 (73)	160 (69.6)
	NO	431 (27)	70 (30.4)
History of abortion	YES	42 (50)	36 (22.5)
	NO	42 (50)	124 (77.5)
Number of abortions	1	27 (64.3)	27 (75)
	≥2	13 (35.7)	9 (25)
History of PROM	YES	32 (38.1)	18 (11.3)
	NO	52 (61.9)	142 (88.7)
History of preterm birth	YES	19 (22.6)	17 (10.6)
	NO	65 (77.4)	143 (89.4)
History of gynecologic surgery	YES	4 (3.5)	10 (4.3)
	NO	111 (96.5)	220 (95.7)
History of cesarean section	YES	18 (21.4)	18 (11.2)
	NO	66 (78.6)	142 (88.8)
Contraceptive use	YES	37 (32.2)	81 (35.2)
	NO	78 (67.8)	149 (64.8)
Type of contraceptive	IUCD	1 (2.2)	6 (7.4)
	Pills	8 (21.6)	30 (37)
	Injectable	16 (43.2)	27 (33.3)
	Implants	10 (27)	15 (18.5)
	Others^a^	2 (5.4)	3 (3.7)

^a^ Natural family planning methods.

The median gestational age of cases and controls were 37 ± 5 IQR (inter quartile range) and 37.7 ± 7 IQR respectively. Among the participants 32 (27.8%) of cases and 67 (29.1%) of controls were primigravidas. The mean birth interval for cases and controls were 31.04 and 31.72 months with SD ± 18.18 and ± 12.91 respectively. Abnormal vaginal discharge was found in 27 (23.5%) cases and 28 (12.2%) of controls. The majority of cases 111 (96.5%) and 217 (94.3%) of controls carried singleton fetus (Table 3).

**Table 3:** Current pregnancy-related characteristics of participants in public hospitals of Harar Town, Eastern Ethiopia 2020 (n = 345; 115 cases and 230 controls).

Variables	Category	Cases (N, %)	Controls (N, %)
Gestational age	<37 weeks	48 (41.7)	44 (19.1)
	37–41 weeks	65 (56.5)	178 (77.4)
	>41 weeks	2 (1.7)	8 (3.5)
Gravidity	One	32 (27.8)	67 (29.1)
	2–4	50 (43.5)	103 (44.8)
	≥5	33 (28.7)	60 (26.1)
Birth interval	<24months	30 (35.7)	41 (25.6)
	≥24 months	44 (64.3)	119 (74.4)
Antenatal visit	Yes	101 (87.8)	199 (86.5)
	No	14 (12.2)	31 (13.5)
Number of visits	One	12 (10.4)	26 (13)
	2–3	27 (26.7)	56 (28)
	>3	62 (62.9)	118 (59)
Pregnancy type	Single-tone	111 (96.5)	217 (94.3)
	Multiple	4 (3.5)	13 (5.7)
Smoking	Yes	3 (2.6)	4 (1.7)
	No	112 (97.4)	226 (98.3)
Khat chewing	Yes	33 (28.7)	58 (25.2)
	No	82 (71.3)	172 (74.8)
Abnormal vaginal discharge	Yes	27 (23.5)	28 (12.2)
	No	88 (76.5)	202 (87.8)
Pre-eclampsia	Yes	2 (1.7)	12 (5.2)
	No	113 (98.3)	218 (94.8)
Abdominal trauma	Yes	9 (7.8)	5 (2.2)
	No	106 (92.2)	225 (97.8)
Lifting heavy object	Yes	19 (16.5)	19 (8.3)
	No	96 (83.5)	211 (91.7)
Fetal presentation	Cephalic	94 (81.7)	204 (88.7)
	Non-cephalic^a^	21 (18.3)	26 (11.3)
Maternal hemoglobin	<11 mg/dl	47 (40.9)	97 (42.2)
	≥11 mg/dl	68 (59.1)	133 (57.8)

^**a**^ Breech presentation, face presentation.

## 6 Determinants of PROM

The odds of PROM among those who had abnormal vaginal discharge were 2 times higher than those who hadn't (AOR = 2.15, 95% CI: 2.53, 22.46). Pregnant women who had a history of cesarean delivery were 2.06 times more likely experiencing PROM than those who had no history of cesarean delivery (AOR = 2.06, 95% CI: 1.11, 6.78). The odds of women with PROM were 4.6 times higher among pregnant women who had a previous history of PROM than their counterparts (AOR = 4.62, 95% CI: 2.06, 11.52). Pregnant women who had a history of abortion were experiencing PROM 2.8 times more likely than those women who hadn't a history of abortion (AOR = 2.81, 95% CI: 1.04, 6.23) (Table 4).

**Table 4:** Bivariate and multivariable analysis for the determinants of PROM in Harar Town public hospitals, eastern Ethiopia 2020 (n = 345).

Variables	Category	Cases	Controls	COR (95% CI)	AOR (95% CI)
Birth interval	<24months	30	41	1.97 (0.13,1.88)	0.363 (0.07,1.94)
	≥24 months	44	119	1	1
History of abortion	YES	42	36	3.44 (1.56, 5.07)	2.81 (1.04, 6.23)*
	NO	42	124	1	1
History of PROM	YES	32	18	4.85 (2.98,11.86)	4.62 (2.06,11.52)**
	NO	52	142	1	1
History of preterm birth	YES	19	17	2.46 (1.54, 6.76)	2.45 (0.83,7.12)
	NO	65	143	1	1
History of C/S	YES	18	18	2.15 (1.05,4.40)	2.06 (1.11, 6.78)*
	NO	66	142	1	1
Abnormal vaginal discharge	Yes	27	28	2.21 (3.76,19.24)	2.15 (2.53, 22.46)*
	No	88	202	1	1
lifting heavy object	Yes	19	19	2.2 (1.11,4.34)	2.01 (0.67, 5.45)
	No	96	211	1	1
Fetal presentation	Cephalic	94	204	1	1
	Non cephalic^a^	21	26	1.75 (0.62,3.04)	0.78 (0.38, 2.62)

^a^ Breech presentation, face presentation, * p-value <0.05 ** p-value <0.001.

## 7 Discussion

This study revealed that abnormal vaginal discharge during the current pregnancy; history of cesarean delivery, history of PROM, and history of abortion were determinants of PROM. The result of this study indicated that mothers who had a previous history of abortion were 2.8 times more likely experiencing PROM than those who hadn't. This finding is consistent with findings reported from Egypt and Northern Ethiopia [8,13]. The possible explanation might be in the case of induced abortions there might be trauma to the lower uterine segment and cervix resulting in weakening of cervical tissues [14,15].

In this study, PROM was 2.06 times higher among women who had a previous history of cesarean delivery than their counterparts. This finding is supported by studies done in Uganda and Tigray [8,16]. This might be due to the concept that cesarean scars could cause abnormal placentation and structural abnormalities in the fetal membrane which could result in tear of membrane and leakage of liquor [16].

Women who had a previous history of PROM were 4.6 times more likely to develop PROM compared to women who had no history of PROM. This finding is supported by study findings from Sweden [17] and Nigeria-Maiduguri [18]. Similarly, a study done in Mekelle-Tigray supports this result [8]. The possible reason for this might be due to the nature of some obstetrical complications to recur in subsequent pregnancies therefore PROM is more likely to occur recurrently [19]. In addition, naturally short cervix length and genital infections might be associated with the outcome [20,21].

Among the present pregnancy-related factors abnormal vaginal discharge was strongly associated with PROM. In this study women who had abnormal vaginal discharge in the current pregnancy were 2 fold more likely to develop PROM than their counterparts. This finding is consistent with study reports in China and Uganda [22,23]. Similar with this study there is also a finding report in Ethiopia that indicates abnormal vaginal discharge as a determinant for PROM. This could be the fact that abnormal vaginal discharge might be related to infection. Infection results in inflammation of the amniotic membrane leading to rupture. There are also genital bacterial proteolytic enzymes (protease, collagenase) that can degrade the fetal membrane resulting in the breakage and rupture of the amniotic sac [22,24,25].

## 8 Strength and limitations

### 8.1 Strength

This study seems one of the pioneer analytical (case control) studies in Harar town, eastern Ethiopia. Data collection with interview is supplemented by card reviews which can minimize missing of data which were difficult to collect through interview.

### 8.1 Limitations

In this study difficult to recall events might be considered as a limitation. In addition, there might be selection bias since case control study was conducted.

## 9 Conclusion

Abnormal vaginal discharge, history of cesarean delivery, history of PROM, and history of abortion were significantly associated with premature rupture of membrane. Planning appropriate pregnancy care during antenatal visits and subsequent follow-up is highly recommended. in addition health care providers are suggested to identify pregnancies which are at risk of developing PROM.

## Recommendations

### Recommendation for Harar regional health bureau

The regional health bureau should strengthen strategies of pregnancy care for women with risky pregnancy such as those with past cesarean delivery, had history of abortion and previous PROM.

### Recommendation for health care providers

Health care providers should timely identify pregnancies with past history of obstetric complications. It is also recommended to provide adequate counseling for mothers on danger signs of pregnancy and their suggestive complications such as abnormal vaginal discharges suggesting infections.

## Author contribution statement

Addisu Getnet: Conceived and designed the concept; Performed the experiment; Analyzed and interpreted the data; Wrote the paper.

Lemessa Oljira, Nega Assefa: Conceived and designed the concept; Contributed reagents, materials, analysis tools or data.

Getahun Tiruye, Zerihun Figa: Analyzed and interpreted the data; Contributed reagents, materials, analysis tools, or data; Wrote the paper.

## Data availability statement

Data will be made available on request.

## Declaration of competing interest

The authors declare no competing interest
